# How to Combine the Two Landmark Treatment Methods—Allogeneic Hematopoietic Stem Cell Transplantation and Chimeric Antigen Receptor T Cell Therapy Together to Cure High-Risk B Cell Acute Lymphoblastic Leukemia?

**DOI:** 10.3389/fimmu.2020.611710

**Published:** 2020-12-15

**Authors:** Mingming Zhang, He Huang

**Affiliations:** ^1^ Bone Marrow Transplantation Center, The First Affiliated Hospital, Zhejiang University School of Medicine, Hangzhou, China; ^2^ Institute of Hematology, Zhejiang University, Hangzhou, China; ^3^ Zhejiang Engineering Laboratory for Stem Cell and Cellular Immunotherapy, Hangzhou, China

**Keywords:** chimeric antigen receptor, acute lymphoblastic leukemia, relapsed or refractory, graft *versus* host disease, minimal residual disease, stem cell transplant

## Abstract

Allogeneic hematopoietic stem cell transplantation (allo-HSCT) has made tremendous progress in the last few decades and is increasingly being used worldwide. The success of haploidentical HSCT has made it possible to have “a donor for everyone”. Patients who received transplantation in remission may have a favorable outcome, while those who were transplanted in advanced stages of disease have a poor prognosis. Although chimeric antigen receptor T (CAR-T) cell therapy is currently a milestone in the immunotherapy of relapsed or refractory (R/R) B cell acute lymphoblastic leukemia (B-ALL) and has demonstrated high remission rates in patients previously treated in multiple lines, the relatively high relapse rate remains a barrier to CAR-T cell therapy becoming an excellent cure option. Therefore, combining these two approaches (allo-HSCT and CAR-T cell therapy) is an attractive area of research to further improve the prognosis of R/R B-ALL. In this review, we will discuss the current clinical practices of combining allo-HSCT with CAR-T cell therapy based on available data, including CAR-T cells as a bridge to allo-HSCT for R/R B-ALL and CAR-T cell infusion for post-transplant relapse. We will further explore not only other possible ways to combine the two approaches, including CAR-T cell therapy to clear minimal residual disease peri-transplantation and incorporation of CAR technology to treat graft-*versus*-host disease, but also the potential of CAR-T cells as a part of allo-HSCT.

## Introduction

Allogeneic hematopoietic stem cell transplantation (allo-HSCT) has achieved great progress in the past few decades. Advances in graft-*versus*-host disease (GVHD) prophylaxis and supportive care have significantly improved the outcomes of allo-HSCT. The success of haploidentical hematopoietic stem cell transplantation (haplo-HSCT) has expanded the application of allo-HSCT, making it possible to have “a donor for everyone”. In recent years, the results of haplo-HSCT have been comparable to HSCT with matched sibling donors and unrelated donors (1–4). As a result, there has been a dramatic increase in the number of haplo-HSCT worldwide (5–7).

However, only transplantation of patients in remission may obtain favorable outcomes, whereas the prognosis of transplantation of patients with advanced disease is poor, with a long-term survival rate of only about 20% (7). Therefore, the efficacy of salvage allo-HSCT for patients with relapsed or refractory (R/R) hematological malignancies is very limited. In addition, post-transplantation relapse still occurs frequently and is the main cause of death after allo-HSCT, yet there is no satisfactory salvage method (8, 9).

The advent of chimeric antigen receptor T (CAR-T) cell therapy offers hope for patients with R/R hematological malignancies. CAR-T cell therapy has shown a high remission rate in these patients with severe pre-treatments (10–19). However, the relatively high relapse rate remains a barrier to CAR-T cell therapy becoming a curable method (10, 11, 20, 21). The integration of allo-HSCT and CAR-T cell therapy becomes an attractive area of research to fully exploit each other’s advantages and further improve the treatment of B-cell malignancies, especially high-risk B-cell acute lymphoblastic leukemia (B-ALL).

To sum up, we will explore the current clinical practices of combined allo-HSCT and CAR-T cell therapy including CAR-T cell therapy as a bridge to allo-HSCT for R/R B-ALL and CAR-T cell infusion for post-transplant relapse, based on available data. And we will also further explore other possible ways to combine the two methods, including the clearance of minimal residual disease (MRD) peri-transplantation by CAR-T cell therapy and the incorporation of CAR technology in the treatment of GVHD. Meanwhile, we will also focus on a number of preclinical or pilot clinical studies targeting for CAR-T cells as part of the graft or conditioning regimen in allo-HSCT.

## Is CAR-T Cell Therapy a Bridge to allo-HSCT or a Definitive Treatment?

The relapse rate of B-ALL after CAR-T cell therapy was 20–70% when the follow-up period was long enough (22). Therefore, it is still controversial whether CAR-T cell therapy is the definitive treatment or bridging therapy to allo-HSCT. Currently, the need for allo-HSCT after CAR-T cell therapy usually depends on the characteristics and persistence of CAR-T cells, the duration of B cell aplasia, institutional experience, and the patient’s intent and general physical condition. For patients who intend to receive allo-HSCT after CAR-T cell therapy, haploidentical donors are an important source of donors due to the rapid donor preparation and the strong effect of graft *versus* leukemia (GVL) (1, 23). **Table 1** presents the results of current large clinical studies of patients requiring allo-HSCT after CAR-T cell therapy. We will discuss pediatric and adult patients separately.

**Table 1:** Summary of large clinical studies related to the need for allo-HSCT after CAR-T cell therapy in B-ALL.

Study	N	Costimulatory domain	Previous HSCT, %	CR/CRi rate, %	MRD- CR rate, %	Allo-HSCT in CR, %	Haplo-HSCT, %	Overall OS, %	Overall RFS/EFS/LFS, %	Allo-HSCT *vs* non- HSCT
Children and young adults										
Maude et al. Phase I/IIA (17)	30	4-1BB	60	90	79	10	NA	78 (at 6 mo)	67 (at 6 mo)	NA
Maude et al. (ELIANA) (15, 24)	79	4-1BB	61	82	81	10	NA	70 (at 18 mo)	66 (at 18 mo)	NA
Gardner et al. (25, 26)	45	4-1BB	62	93	93	28	NA	69 (at 12 mo)	51 (at 12 mo)	LFS, P = 0.057
Lee et al. (27–29)	51	CD28	35*	61	55	75	NA	52 (at 10 mo)*	49 (at 18 mo)	Relapse (9 *vs* 86%, P = 0.001); LFS, P = 0.006
Zhang et al. (30)	110 (65% children)	4-1BB (81%) CD28 (19%)	14	93	87	73	67	64 (at 12 mo)	58 (at 12 mo)	LFS (77 *vs* 11%, P < 0.0001); OS (79 *vs* 32%, P < 0.0001)
Adults										
Park et al. (10)	53	CD28	36	83	67	39	NA	50 (at 13 mo)	50 (at 6 mo)	EFS, P = 0.64; OS, P = 0.89
Jiang et al. (31)	58 (5 children)	4-1BB	5	88	81	45	62	61 (at 12 mo)	50 (at 7.3 m)	RFS, P = 0.001; OS, P = 0.099
Turtle et al. (32, 33)	53	4-1BB	43	85	85	40	0	50 (at 20 mo)†	50 (at 7.6 mo)†	EFS (HR = 0.39 P = 0.088)
Gu et al. (34)	56 (Ph+ ALL)	4-1BB	0	91	68	59	83	50 (at 16 mo)	50 (at 15 mo)	OS (59 *vs* 23%, P = 0.005); EFS (53 *vs* 19%, P < 0.001)
Zhao et al. (35)	122	4-1BB	20	100	100	45	100	NA	NA	LFS, P < 0.001; OS, P < 0.001

HSCT, hematopoietic stem cell transplantation; CR, complete remission; CRi, complete remission with incomplete count recovery; MRD, minimal residual disease; Allo-HSCT, allogeneic HSCT; Haplo-HSCT, Haploidentical HSCT; OS, overall survival; RFS, relapse-free survival; EFS, event-free survival; LFS, leukemia-free survival.

*Results were reported from the first 21 patients.

†The authors reported survival rates in patients achieving MRD negative CR after CAR-T cell therapy.

For pediatric and young adult patients with R/R B-ALL, a phase 1/2a study involved 30 patients treated with CD19 CAR-T cell therapy. After CAR-T cell therapy, only 10% of patients underwent allo-HSCT. Despite the low percentage of subsequent allo-HSCT, the event-free survival (EFS) rate was 67%, and the overall survival (OS) rate was 78% at 6 months of continuous remission (17). Subsequently, a global phase 2 study of Tisagenlecleucel in 75 patients showed that only eight patients in remission underwent allo-HSCT (15). The EFS and OS rates at 12 months were 50 and 76%, and the median duration of remission was still not reached after a median follow-up of 13.1 months. In both studies, the persistence of CAR-T cells and the duration of B cell aplasia were long.

In contrast, a phase 1 study at Seattle Children’s Hospital enrolled 45 children and adolescents with R/R B-ALL in CD19 CAR-T cell therapy. The MRD-negative complete remission (CR) rate was 93%, but the median expected duration of B cell aplasia was only 3 months. Of the 40 patients with MRD-negative CR, 11 (27.5%) underwent consolidative allo-HSCT, and only two (18%) patients experienced relapse after allo-HSCT. Of the 29 patients who did not undergo consolidative allo-HSCT, 16 patients (55%) relapsed with a median follow-up of 12.2 months (25). Another study from Pediatric Oncology Branch of the National Cancer Institute enrolled 20 children and young adults with R/R B-ALL who received a single infusion of CD28-containing anti-CD19 CAR-T cells (27). A total of 12 patients achieved MRD-negative CR. The persistence of CAR-T cells was relatively short, and no CAR-T cells were detected after day 68. Thus, a high proportion (83%) of patients who obtained MRD-negative CR underwent subsequent allo-HSCT. All 10 patients who underwent allo-HSCT remained disease-free, and no unexpected peri-transplant toxicity was observed. Two patients were judged ineligible to undergo allo-HSCT and both relapsed within a short time (27). In a recent large phase 1/2 study from China, a total of 110 patients with B-ALL were infused with CD19 CAR-T cells (30). The majority of patients were children. Morphologic CR was observed in 93% of patients, and 87% achieved MRD negativity. 75 patients (73.5%) subsequently received allo-HSCT and 50 patients received haplo-HSCT. Leukemia-free survival (LFS, 76.9 *vs* 11.6%, P<0.0001) and OS (79.1 *vs* 32.0%, P < 0.0001) were significantly better in patients who underwent allo-HSCT compared with those who received only CAR-T cell therapy. The authors speculated that in the majority of the patients, haplo-HSCT (67%) and a myeloablative conditioning regimen may play a role to reduce leukemia relapse.

For adults with R/R B-ALL, a phase 1 trial from MSKCC first reported the results of patients receiving 19-28z CAR-T cell therapy (10). A total of 53 adults were enrolled and 44 (83%) patients achieved CR. Among the 44 patients with CR, 17 (39%) patients proceeded to allo-HSCT. There was no significant difference in EFS and OS between MRD-negative patients who underwent allo-HSCT and those who did not. A clinical trial from China included 53 adults and five pediatric R/R B-ALL patients who received CD19 CAR-T cell therapy (31). Of the 47 patients with MRD-negative remission, 21 were bridged to allo-HSCT. Overall, no difference was found in OS between patients who received allo-HSCT and those who did not. However, the trial further identified subgroups of patients with high (≥5%) pre-infusion bone marrow MRD or poor prognostic markers and found that only this subgroup benefited from allo-HSCT with significantly prolonged EFS.

On the contrary, in a phase 1/2 clinical trial from Fred Hutchinson Cancer Research Center, 45 (85%) of the 53 patients who received CD19 CAR T-cell therapy achieved MRD-negative CR. Eighteen (40%) patients in MRD-negative CR underwent allo-HSCT. Multivariable stepwise modeling demonstrated that allo-HSCT after CAR-T cell therapy may achieve a better EFS (32, 33). Gu B et al. reported a study of adults with R/R Philadelphia-chromosome positive ALL receiving humanized CD19 CAR-T cell therapy. Fifty-one/56 (91.1%) patients achieved CR or CR with inadequate count recovery (CRi). Subsequently, 30/51 CR/CRi patients received consolidative allo-HSCT. Patients with allo-HSCT had better 2-year OS and LFS than those without allo-HSCT. Multivariable analysis revealed that allo-HSCT and MRD-negative remission were independent prognostic factors of OS and LFS (34). Recently, we conducted a multicenter retrospective study to assess whether patients can benefit from haplo-HSCT after CAR-T cell therapy or not (35). A total of 122 patients were enrolled, including 55 patients with subsequent haplo-HSCT and 67 patients without subsequent transplantation. Compared to the non-transplant group, patients who received subsequent haplo-HSCT had higher 2-year OS (77.0 *vs* 36.4%, P < 0.001) and LFS (65.6 *vs* 32.8%, P < 0.001). In addition, MRD-negativity before transplantation predicts a favorable outcome of CAR-T cell therapy followed by haplo-HSCT.

From the above findings, the need to bridge allo-HSCT after R/R B-ALL remission with CAR-T cell therapy is still a controversial topic. **Table 2** lists the ongoing clinical trials of CAR-T cell therapy bridging to allo-HSCT in the treatment of B cell malignancies. Bridging allo-HSCT, while reducing relapse rates, is associated with transplant-related mortality. The most critical factor for the future will be the identification of risk factors for relapse after CAR-T cell therapy and selective bridging of allo-HSCT in high-risk patients. For patients with a low risk of relapse after CAR-T cell therapy, close monitoring is all that needed.

**Table 2:** Ongoing clinical trials of CAR-T cell therapy bridging to allo-HSCT in the treatment of B cell malignancies.

Trial ID	Phase	Disease	Disease status	Target	Estimated enrollment	Conductor
NCT03366324	1/2	B-cell Malignancies	MRD positive	CD19	20	Union Hospital, Tongji Medical College, Huazhong University of Science and Technology, China
NCT03366350	1/2	B-cell Malignancies	R/R	CD19	50	Union Hospital, Tongji Medical College, Huazhong University of Science and Technology, China
NCT04626726	1/2	B-ALL	R/R	CD19/CD22	50	No.2 Hospital of Hebei Medical University, China
NCT02846584	2	B-cell Malignancies	R/R	CD19/CD20	100	Southwest Hospital of Third Military Medical University, China
NCT03110640	1	B-cell Leukemia/Lymphoma	R/R	CD19	20	The First Affiliated Hospital of Wenzhou Medical University, China
NCT02431988	1	Diffuse Large B Cell Lymphoma	R/R	CD19	10	University College London Hospital, London, United Kingdom

B-ALL, B cell acute lymphoblastic leukemia; MRD, minimal residual disease; R/R, relapsed or refractory.

## CAR-T Cell Therapy to Treat Post-Transplant Relapse With Low Incidence of GVHD

Relapse is the leading cause of death after allo-HSCT (36). The prognosis of relapse after allo-HSCT is very dismal, with low remission rates and poor long-term survival (37, 38). The median survival after relapse is 5.5 months. The estimated survival rates at 1-, 2- and 5-year after relapse are 30, 16, and 8%, respectively (9). Despite the development of allo-HSCT for the decades, the treatment of relapse after allo-HSCT remains a major challenge. Augmentation of the GVL effect through donor lymphocyte infusion (DLI) is one of the major salvage interventions for post-transplant relapse (39–43).

However, DLI has a limited effect on ALL relapse after allo-HSCT, with a CR rate of only 27% (44). Moreover, the application of DLI is limited by the development of acute or chronic GVHD (40–60%) (45, 46). Therefore, new therapeutic strategies are urgently needed to improve the prognosis of ALL relapsed after allo-HSCT. CAR-T cell therapy has brought revolutionary progress in the treatment of R/R hematological malignancies. At present, CAR-T cells still show great potential in the treatment of post-transplant relapse. T cells harvested for CAR-T preparation may come from donors or recipients (**Table 3**).

**Table 3:** Clinical outcomes of CAR-T cell therapy for post-transplant relapse.

Study	N	Costimulatory domain	CR/CRi rate, %	Acute GVHD, %	Chronic GVHD, %
Donor derived allogeneic CAR-T cells					
Kochenderfer et al. (47, 48)	20	CD28	80*	0	10
Cruz et al. (49)	8	CD28	50^†^	0	0
Dai et al. (50)	2	4-1BB	50	100 (grade 2 to 3)	0
Hu et al. (51)	3	4-1BB	67	33.3 (grade 3)	NA
Recipient derived allogeneic CAR-T cells					
Park et al. (10)	19	CD28	84	0	0
Maude et al. (17)	18	4-1BB	NA	0	0
Lee et al. (27)	7	CD28	57	0	0
Zhang et al. (30)	16	4-1BB^‡^	94	12.5 (grades 1 and 3)	12.5
Hu et al. (51)	11	4-1BB	100	18.2 (grade 2)	NA
Turtle et al. (32)	11	4-1BB	93	0	9
Gardner et al. (25)	27	4-1BB	93	3.7 (grade 3)	0

CR, complete remission; CRi, complete remission with incomplete count recovery; GVHD, graft versus host disease.

*CR rate was calculated from five ALL patients.

^†^CR rate was calculated from two relapsed ALL patients.

^‡^81% of 110 enrolled patients received 4-1BB costimulatory CAR-T cells.

For the first time, Kochenderfer et al. infused donor-derived allogeneic CD19 CAR-T cells into patients with malignancies that persisted after allo-HSCT and standard DLI (47, 48). CAR-T cells were infused without previous chemotherapy or lymphocyte depletion conditioning. Eight of 20 patients with B-cell malignancies obtained remission, which included six CRs and two partial remissions. B-ALL had the highest response rate, with four of five patients achieving MRD-negative CRs. In another study, Cruz et al. reported a phase one study in which donor-derived virus-specific T cells were engineered to express CD19 CAR. CR was achieved in one of two patients with B-ALL relapsing after allo-HSCT (49). In our report, two of three patients (66.7%) with relapsed B-ALL post-transplantation obtained CR after receiving donor-derived CD19 CAR-T cell therapy (51).

In addition to donor-derived T cells, CAR-T cells can also be manufactured from T cells harvested from the recipients. In several studies described in the previous chapters (10, 17, 27), patients with R/R B-ALL who relapsed after allo-HSCT were also included. The reported CR rates after CAR-T cell therapy ranged from 57 to 84%. In our study (51), we included 11 patients who received recipient-derived CAR-T cell therapy for post-transplant relapse. All patients (100%) achieved CR after CAR-T cell therapy. In another study from China, efficacy of CD19 CAR-T cell in high-risk B-ALL was evaluated (30). Sixteen patients had allo-HSCT prior to CAR-T cell therapy, and 11 (68.8%) had at least one DLI. After CAR-T cell therapy, 15 (93.8%) patients achieved CR. No statistically significant difference was observed in the rate of CR in patients who received allogeneic or autologous CAR-T cell therapy.

From the above data, CAR-T cell therapy has good efficacy in the treatment of post-transplant relapse. In addition to the routine complications such as cytokine release syndrome (CRS) and immune effector cell-associated neurotoxicity syndrome (ICANS), allogeneic CAR-T cells infusion brings concerns about GVHD induction. In the study from Kochenderfer et al. (47, 48), a total of 14 patients had a history of GVHD, but none developed new-onset acute GVHD after CAR-T cell infusion. One patient developed mild chronic ocular GVHD 2 years later, and another patient had chronic GVHD at study entry, but the disease slowly and progressively worsened. In the study by Cruz et al. (49), no GVHD was observed after donor-derived CAR-T cell therapy, whereas we observed that acute GVHD in one of three patients following donor-derived CAR-T cell infusion. This patient was diagnosed with grade 3 gastrointestinal GVHD with secretory diarrhea more than 10 times per day. Symptoms improved after combination therapy with steroids, cyclosporin, mycophenolate, and ruxolitinib (51).

For recipient-derived CAR-T cell therapy, Park et al. (10), Maude et al. (17), and Lee et al. (27) reported a total of 43 cases but no GVHD was observed. Two studies from China showed that a small proportion of patients experienced GVHD after CAR-T cell infusion. One study showed that out of 16 patients, two (12.5%) patients developed acute GVHD (grade 1 and grade 3), and two (12.5%) patients developed extensive chronic GVHD (30). In our report, two of 11 patients (18.2%) developed grade 2 acute skin GVHD after infusion of recipient-derived CAR-T cells (51).

For GVHD caused by allogeneic CAR-T infusion, it is unclear whether treatment of GVHD affects the persistence and effectiveness of CAR-T cells. In a pilot study, two B-ALL patients received donor-derived 4-1BB costimulatory CAR-T cell therapy after allo-HSCT and developed grades 2–3 acute GVHD 3–4 weeks after cell infusion. Symptoms of GVHD were easily relieved with short-term use of steroids and/or cyclosporin A. However, after anti-GVHD therapy, one patient with moderately reduced blasts in bone marrow rapidly progressed and died, and another patient with hematologic CR achieved CD19 positive relapse (50). Nevertheless, a recent case report presented that allogeneic donor-derived 4-1BB based CAR-T cells were persistent up to 6 months after infusion under therapeutic levels of cyclosporine A (52).

In contrast to the aforementioned studies using CAR-T cells prepared from unselected T cells, two studies engineered 4-1BB containing CAR-T cell products, which consisted of a defined 1:1 ratio of CD4+: CD8+ CAR-T cells (25, 32). This highly defined CD19 CAR T-cell product was remarkably potent, with over 90% of patients achieving CR after CAR-T cell therapy in both studies. Turtle et al. reported that 27 (93%) of 29 patients with R/R B-ALL achieved bone marrow remission after CAR-T cell therapy. Patients who received lymphodepletion with fludarabine and cyclophosphamide before CAR-T cell therapy achieved a 1-year DFS rate greater than 60%. Eleven patients with prior allo-HSCT received infusions of CAR-T cells manufactured from recipients. None of the 11 patients developed acute GVHD after CAR-T cell therapy. One patient who had grade 1 acute skin GVHD before study enrollment developed chronic GVHD at 3 months after CAR-T cell infusion and required corticosteroid therapy (32). In another study of 45 patients with R/R B-ALL, the MRD negative remission rate after CAR-T cell therapy was 93%. The estimated 12-month EFS of the infused patients was 50.8%, the estimated 12-month OS was 69.5%, and the median follow-up time was 9.6 months. Twenty-seven patients in this study had undergone prior allo-HSCT. One patient had a history of GVHD, which had been phased off GVHD medication for more than 1 year prior to CAR-T cell therapy, and developed grade 3 acute skin GVHD (25).

Compared with DLI, CAR-T cell therapy has a higher remission rate for post-transplant relapse and the incidence of GVHD associated with CAR-T cells infusion seems to be relatively low. To date, a summary of all data on CAR-T cell therapy for post-transplant relapse showed that the incidence of GVHD was less than 10%. The risk factors for allogeneic CAR-T cell-associated GVHD have not been fully defined. But from the current data, it may be related to the source of T cells (donor- or recipient-derived), CAR structure (53–56), CAR-T cell subpopulation, the history of GVHD after allo-HSCT, which needs to be further clarified by larger data support.

## CAR-T Cell Therapy to Clear Peri-Transplantation MRD

CAR-T cell therapy improves the outcomes of R/R ALL strikingly, but has potentially life-threatening complications, including CRS and ICANS, especially in patients with high disease burdens. Although most patients make a full recovery after treatment, patients with grades 3 to 4 CRS or ICANS are recommended to be transferred to the intensive care unit, and a small percentage of patients still die because of serious complications. Therefore, CAR-T cell therapy could be used more safely to clear MRD with morphological remission, which is suggested to accompany mild complications. In addition, MRD is a powerful prognostic factor in the treatment of ALL (57–63). For ALL patients receiving allo-HSCT, peri-transplantation MRD levels have been confirmed to be significantly associated with post-transplant relapse and long-term survival. Thus, for B-ALL patients undergoing allo-HSCT, the application of CAR-T cell therapy to clear peri-transplantation MRD is an effective and safe way to improve the prognosis. Previous studies on CAR-T cell therapy included patients with MRD-positive remission and patients with elevated MRD after transplantation.

Park et al. included 15 patients who had MRD with bone marrow blasts rates ranging from 0.01 to <5% and six patients with MRD-negative remission (10). Results showed that when compared with higher disease burden (≥5% bone marrow blasts), lower disease burden (<5% bone marrow blasts) was associated with a lower risk in severe CRS (41 *vs* 5%, P = 0.004) and neurotoxic effects (59 *vs* 14%, P = 0.002). Moreover, patients with lower disease burden had significantly longer EFS (10.6 *vs* 5.3 months, P = 0.01) and OS (20.1 *vs* 12.4 months, P = 0.02) than patients with higher disease burden. But there was no significant difference in survival between patients with lower disease burden who underwent transplantation and those who did not.

Another study included six patients with marrow blasts less than or equal to 5%, two of whom were MRD-positive after transplantation (27). Patients with higher disease burden were significantly more likely to have grades 3 or 4 CRS than patients with lower disease burdens (P = 0.039). After CAR-T cell therapy, all six patients obtained MRD-negative remission. Five of them underwent subsequent allo-HSCT after MRD clearance and remained disease-free with no unexpected peri-transplant toxicities. One patient with previous allo-HSCT was ineligible to receive a second allo-HSCT and relapsed with CD19-negative leukemia 3 months later.

In a study of 110 high-risk ALL patients treated with CAR-T cell therapy, 42 patients with MRD-positive remission were included (30). CAR-T cell therapy successfully cleared MRD in all 42 patients with a significantly lower incidence of grades 3 to 4 CRS and grades 2 to 3 neurotoxicity compared with patients who had morphologic relapse. The majority of patients (73.5%) in this study received subsequent allo-HSCT and achieved an LFS of 76.9% at 1 year. Notably, among the 75 patients who received allo-HSCT, only seven (10.1%) of 69 MRD-negative patients relapsed after transplantation, while three (50%) of six MRD-positive patients relapsed after transplantation. This reflected the importance of clearing MRD before transplantation to reduce post-transplant relapse.

Kebriaei et al. conducted a phase 1 trial in 17 B-ALL patients who received allogeneic CD19 CAR-T cells infusion to target MRD at a median of 64 days after allo-HSCT (64). CAR T cells were administered without additional lymphodepletion. GVHD prophylaxis was tapered and discontinued by 6 months after allo-HSCT. No unexpected acute infusion or delayed toxicities were noted. Three patients developed GVHD, one patient with grade one acute skin GVHD and one patient with chronic skin GVHD who responded to steroids. One patient with a prior history of drug-induced hepatotoxicity died from hepatic GVHD. Following allo-HSCT, 1-year PFS and OS were 53 and 63%, respectively. When the subset of patients who received haplo-HSCT was analyzed, the respective1-year rates were 75 and 100%, respectively. In a similar study, Zhang C et al. reported that two high-risk ALL patients who received haplo-HSCT were prophylactically infused with donor CAR-T cells on day 60 without CRS and GVHD. Two patients survived with disease-free for 1 year and 6 months, respectively (65).

From the above results of the studies, CAR-T cell therapy is an effective and safe method to clear peri-transplantation MRD. At present, there are an increasing number of clinical studies in this field. As more studies confirm the results, the clearance of MRD will greatly expand the application of CAR-T cell therapy. In addition, whether prophylactic CAR-T cells infusion for high-risk ALL with MRD-negative remission can prevent relapse is another interesting topic.

## Incorporation of CAR Technology Into the Treatment of GVHD

GVHD is the most frequent complication after allo-HSCT (66, 67). Despite improvements in post-transplant immunosuppression, 20–60% of recipients still develop GVHD, which is the leading cause of non-relapse mortality following allo-HSCT (7). Alloreactive T cells mediated immune injury to the host organ is a key process in GVHD. Therefore, negative regulation of T cells to induce immune tolerance is the main method to prevent and treat GVHD. In recent decades, the commonly used immunosuppressive agents for GVHD include steroids, calcineurin inhibitors, and mycophenolate mofetil, *etc*. However, due to the lack of specificity of these drugs and the requirement of long-term maintenance, they can lead to loss of T cell immune function, weaken the anti-infection and anti-leukemic effects of T cells after allo-HSCT, and increase the risk of infection and relapse.

In recent years, an increasing subpopulation of immune cell have been considered to play a role in GVHD (68). Adoptive transfusion of immune cells in GVHD has attracted increasing attention. Previous studies have shown that regulatory T cells (Tregs) infusion can prevent and treat GVHD effectively and have little influence on GVL effects (69–73). Other immune cell subsets, such as NK cells, NKT cells, myeloid derived suppressor cells and type II innate lymphocytes, have also been proved to reduce the incidence of GVHD in a series of preclinical and clinical studies, while the GVL effect remains (74–79).

However, a large number of polyclonal Tregs infusion without antigen specificity leads to widespread, non-specific immunosuppression. Compared with polyclonal Tregs, antigen-specific Tregs have the advantage of migrating to target antigen, persisting in local tissues and mediating local immunosuppression (80, 81). Thus, a relatively small number of antigen-specific Tregs will be sufficient to produce immunosuppression (80, 82). Antigen-specific Tregs can be enriched from alloreactive T cells following stimulation with allogeneic antigen-presenting cells *in vitro*. The expansion efficiency *in vitro* is relatively low, which can limit the number of cells and their universal application in patients. In addition, the extensive expansion of antigen-specific Tregs by antigen-presenting cells stimulation will lead to loss of FOXP3 (83) and decreased survival *in vivo* (84).

The emergence of CAR technology enables T cells to specifically recognize, bind and clear targeted cells in a non-MHC restricted manner. These characteristics of CAR technology have opened new ideas for conferring Treg cell specificity, or CAR-Tregs. CAR-Tregs have a stable phenotype and function without MHC restriction and are less dependent on IL-2. It preferentially migrates to target sites and has stronger specific immunosuppressive effects (85). In animal models, CAR-Treg has shown great potential in the treatment of various diseases, especially autoimmune diseases (86–90).

MHC class I molecules are constitutively expressed on the surface of almost all nucleated cells and are major determinants of allo-HSCT compatibility. Therefore, MHC class I molecules are potential target antigens for CAR-Tregs to induce immune tolerance after allo-HSCT. In 2016, a group created HLA-A2–specific CAR and its application in generating antigen-specific Tregs (91). *In vitro*, A2-CAR-Tregs maintained their expected phenotype and inhibitory function before, during, and after A2-CAR-mediated stimulation and did not have cytolytic activity. In a mouse model of xenogeneic GVHD transplanted from human PBMCs to NSG mice, human A2-CAR-Tregs were superior to Tregs expressing unrelated CAR in preventing xenogeneic GVHD caused by HLA-A2+ T cells. Two other groups also established A2-CAR-Tregs and demonstrated their enhanced inhibitory function in a human skin xenograft transplant model (92, 93). More recently, Dawson et al. developed a panel of humanized A2-CARs and tested them in Tregs. Adoptive transfer of humanized A2-CAR Tregs *in vivo* showed that humanized A2-CAR Tregs migrate rapidly and persist in A2-expressing allografts, suppress HLA-A2+ cell-mediated xenogeneic GVHD, and diminish rejection of human HLA-A2 + skin allografts (94).

Besides cell-based immunosuppression, another strategy to control GVHD is to target important cells or molecules in the process of GVHD. CD83 is an important marker to define activated human dendritic cells. CD83 is also expressed on activated human T lymphocytes, but not on natural Treg (95). Previous studies have shown that monoclonal antibodies targeting CD83 can reduce GVHD in mice without affecting GVL and antiviral activity (96). Therefore, CD83 may be a potential target for CAR-T cells for the prevention and treatment of GVHD. As mentioned above, CAR-T cells have the property of recognizing, binding, and clearing cells carrying target antigens and infusion of donor-derived CAR-T cells after allo-HSCT is less likely to elicit GVHD. Based on these characteristics of CAR-T cells, human CD83-targeted CAR-T cells have been developed for the prevention of GVHD (97). Human CD83 CAR-T cells can eradicate pathogenic CD83+ target cells, substantially increase the ratio of Tregs to allo-activated conventional CD4+ T cells, and have preventive and therapeutic effects on xenogeneic GVHD.

## Allogeneic CAR-T Cells as Part of HAPLO-HSCT

For patients with high leukemia burden, it is difficult to collect enough autologous T cells in CAR-T cell production. There are also cases where the autologous T cells fail to produce CAR-T cells due to T cell dysfunction and the effects of previous chemotherapy. Allogeneic CAR-T cells may solve this problem. However, allogeneic CAR-T cells will be quickly eliminated by the patient’s immune system without additional gene editing or long-term lymphodepletion.

Two groups from China developed a new method to co-infuse allogeneic CAR-T cells with allogeneic hematopoietic stem cells from haploidentical donor into R/R B-ALL patients (98–100). After re-induction of chemotherapy or a reduced-intensity conditioning regimen, haploidentical donor-derived CD19-CAR-T cells were infused in incremental numbers for 4 days. Haploidentical hematopoietic stem cells were infused after CAR-T cells infusion. The infusion of CAR-T cells as part of the conditioning regimen eradicated leukemia cells and the patients’ normal B cells, and may improve hematopoietic stem cells engraftment. In turn, engraftment of allogeneic hematopoietic stem cells can further enhance the amplification and persistence of allogeneic CAR-T cells. A total of 4 patients with R/R B-ALL were reportedly treated with this protocol. An MRD-negative remission was achieved and complete donor cell engraftment was established. One patient did not have GVHD because of GVHD prophylaxis, but had a short duration of CAR-T cells persistence. The remaining three patients without GVHD prophylaxis developed varying degrees of GVHD, but the CAR-T cells persist relatively longer with the longest persistence up to 20 months. Two patients died from severe infections and two patients survived for 100 days and 20 months with disease-free, respectively.

Recently, Wiebking et al. designed an intriguing approach which combined both allo-HSCT and CAR-T cell therapy with complementary anti-leukemia mechanisms: the HLA-dependent activity of GVL effect and the HLA-independent mechanism of CAR-T cell (101). In this setting, a TCR*αβ*/CD19-depleted haplo-HSCT platform was employed, which was associated with very low transplantation-related mortality and GVHD incidence (102–105). CAR-T cells were manufactured from depleted *αβ* T cells by genome editing to express CD19-specific CARs, while simultaneously inactivating the T cell receptor and rejoining the graft of haplo-HSCT. *In vivo*, the *αβ*TCR-CD19 CAR-T cells eliminated leukemia without causing GVHD in a preclinical xenograft model. This appealing program needs to be further verified in the clinical setting.

## Conclusions

**Figure 1:**
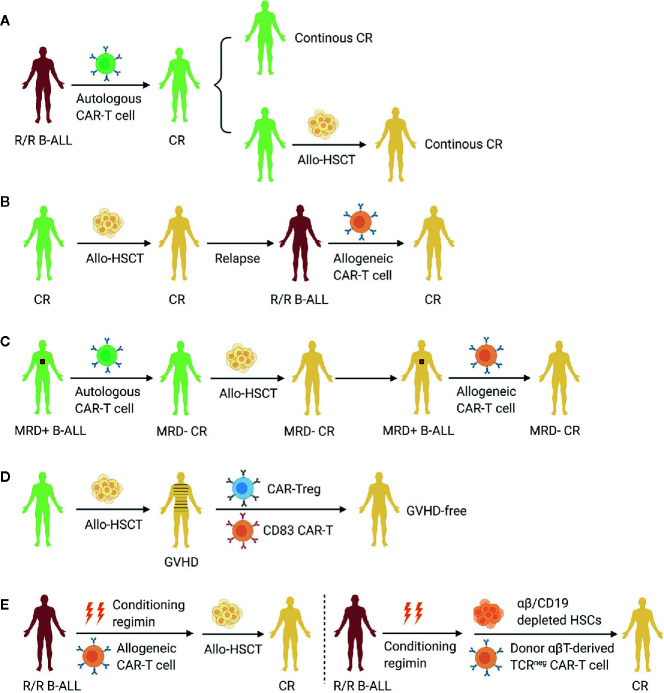
Allo-HSCT in combination with CAR-T cell therapy aiming to improve the prognosis of B-ALL. **(A)** CAR-T cell therapy as a definitive treatment or a bridge to allo-HSCT for R/R B-ALL. **(B)** Infusion of allogeneic CAR-T cells to treat post-transplant relapse. **(C)** Clearance of minimal residual disease peri-transplantation by CAR-T cell therapy. **(D)** Incorporation of CAR technology into the treatment of GVHD. **(E)** CAR-T cells as part of the conditioning regimen or graft in allo-HSCT. R/R B-ALL, relapsed or refractory B cell acute lymphoblastic leukemia; CR, complete remission; Allo-HSCT, allogeneic hematopoietic stem cell transplantation; CAR, chimeric antigen receptor; MRD, minimal residual disease; GVHD, graft-versus-host disease; Treg, regulatory T cell.

The treatment of high-risk ALL remains a challenging. Especially for adult ALL, the outcomes of receiving chemotherapy alone are still poor (106). The establishment of the haplo-HSCT system, which allows almost all patients to have a donor, has greatly improved the prognosis of ALL. The emergence of CAR-T cell therapy has further brought an amazing breakthrough in the treatment of R/R B-ALL. At present, the two therapeutic approaches (allo-HSCT and CAR-T cell therapy) have their own indications and mechanisms, which are difficult to be completely replaced. Combining the two approaches to establish a complete B-ALL treatment system will become an important development area at present and in the future, so as to further improve the prognosis of B-ALL and approach the goal of curing B-ALL (**Figure 1**). According to the available data, CAR-T cell therapy can obtain a high remission rate in R/R B-ALL patients. After remission, some patients can obtain long-term CAR-T cells persistence and disease-free survival, which makes CAR-T cell therapy a definitive method, while other patients need subsequent allo-HSCT to further reduce relapse rates. For B-ALL patients with post-transplant relapse, infusion of allogeneic CAR-T cells also achieves high remission rates with low incidence of GVHD. It is not clear whether secondary transplantation is necessary or not according to the small number of cases. Haplo-HSCT is suggested to be associated with higher incidence of GVHD compared with allo-HSCT from matched sibling donors. CAR technology is a good strategy for the treatment of GVHD. The results from preclinical studies are encouraging and its clinical application is worth expectation in the future. In addition, CAR-T cells are also being explored as a part of haplo-HSCT, such as conditioning regimen or graft, and the complementary mechanism of the two methods are expected to bring better therapeutic effect.

## Author Contributions

HH and MZ designed the structure of the paper. MZ wrote this paper. All authors contributed to the article and approved the submitted version.

## Funding

This work was supported by the National Natural Science Foundation of China (81800178).

## Conflict of Interest

The authors declare that the research was conducted in the absence of any commercial or financial relationships that could be construed as a potential conflict of interest.
